# Diagnosing rare diseases after the exome

**DOI:** 10.1101/mcs.a003392

**Published:** 2018-12

**Authors:** Laure Frésard, Stephen B. Montgomery

**Affiliations:** 1 Department of Pathology, Stanford University, Stanford, California 94305, USA;; 2 Department of Genetics, School of Medicine, Stanford, California 94305, USA

## Abstract

High-throughput sequencing has ushered in a diversity of approaches for identifying genetic variants and understanding genome structure and function. When applied to individuals with rare genetic diseases, these approaches have greatly accelerated gene discovery and patient diagnosis. Over the past decade, exome sequencing has emerged as a comprehensive and cost-effective approach to identify pathogenic variants in the protein-coding regions of the genome. However, for individuals in whom exome-sequencing fails to identify a pathogenic variant, we discuss recent advances that are helping to reduce the diagnostic gap.

High-throughput sequencing has helped to uncover the rates at which deleterious or damaging alleles accumulate in specific genes (Lek et al. 2016). For patients with shared rare diseases, it has aided in identifying novel causal genes by their excess of pathogenic variants (Ng et al. 2010; Muona et al. 2015; Lelieveld et al. 2016; Deciphering Developmental Disorders Study 2017). In clinical settings, depending on disease type and patient selection, exome sequencing has been estimated to lead to a diagnosis in 30%–50% of rare Mendelian diseases (McInerney-Leo et al. 2013; Veeramah et al. 2013; Clark et al. 2018). However, diagnoses of diseases involving novel mechanisms (Oláhová et al. 2018) can be more challenging to perform than well described—although rare—pathologies (Aartsma-Rus et al. 2016). For “exome-negative” cases, in which no such diagnosis is provided, there remain multiple approaches after the exome—but choosing that next step in an “experimental maze” still remains a major challenge.

Negative exome results can be explained in various ways (Fig. 1). To summarize, the causal variant can be missed in cases of somatic mosaicism (Priest et al. 2016): If it is in coding regions but not properly detected (Cornish and Guda 2015); if it is of unknown significance and was not selected (McCarthy et al. 2014; Hoffman-Andrews 2017); if multiple variants are responsible for the pathology, in two or more genes (Liu et al. 2007; Sambuughin et al. 2018); if it is a structural variant not properly caught through exome sequencing (Merker et al. 2018); and in cases in which it is not exonic (Short et al. 2018).

**Figure 1.**
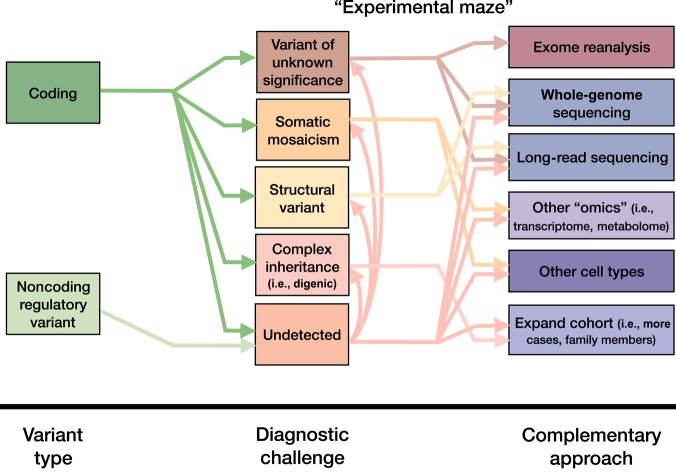
Challenges and approaches in “exome-negative” cases. Depending on the causal variant type, different options are available, influenced by both technical and biological diagnostic challenges. Without knowing the cause of the disease, it can be challenging to select a complementary approach in the postexome “experimental maze.”

Without prior biological knowledge, exome sequencing a single case has a low probability of yielding a novel pathogenic variant or gene. The average individual carries approximately 20 rare, “loss-of-function” variants (MAF ≤ 0.1) (Dewey et al. 2016; Lek et al. 2016) among which four are splice disrupting. Subsequent filtering of these candidate variants to identify disease genes is generally easier for recessive inherited diseases than dominant inherited diseases in which one causal variant is expected instead of two. However, in both scenarios, identifying which variants are most likely to be pathogenic has required either large control data sets (Dewey et al. 2016; Lek et al. 2016) or family-based sequencing data such as trio sequencing, particularly in the context of de novo variant discovery (Zhu et al. 2015; Wang et al. 2016; Jin et al. 2017; Wright et al. 2018a). Hence, one of the most active areas of development has been the ongoing expansion and development of large exome resources. Databases such as ExAC or gnomAD (Lek et al. 2016) and DiscoverEHR (Dewey et al. 2016) currently provide data on more than 100,000 exomes from diverse populations combined. Using the ExAC database, Lek et al. developed the pLI score that reflects each gene's intolerance to loss-of-function variation. This score provides priors on the likelihood of seeing impactful variants in specific genes within a healthy population sample and has been particularly informative for discovery of genes contributing to dominant Mendelian disorders (Eilbeck et al. 2017; Rao and Nelson 2018). Recently, Coban-Akdemir et al. (2018) developed an NMD escape intolerance score to identify genes in which nonsense alleles may contribute to gain of function. As these exome resources grow, new and refined scoring metrics that account for different forms of inheritance and variant impacts are expected to provide new opportunities for variant interpretation.

Beyond enhancing sample sizes of case and controls, additional biological knowledge that aids in identifying candidate causal genes takes advantage of ongoing annotation of gene and phenotype relationships. Extensive and accurate phenotyping is essential to establish a proper link between potential candidate genes and disease characteristics. Tools such as DECIPHER (Firth et al. 2009), the Matchmaker Exchange (Philippakis et al. 2015), GeneMatcher (Sobreira et al. 2015), or PhenomeCentral (Buske et al. 2015) are allowing to match cases with similar phenotypes and/or genotypic profiles to help the diagnosis of rare diseases. Databases such as Human Phenotype Ontology (HPO; Köhler et al. 2017), OMIM (Hamosh et al. 2005), and Orphanet (INSERM 1997) provide useful information to narrow down candidate genes. HPO links phenotypes to genes and diseases. OMIM (https://www.ncbi.nlm.nih.gov/omim/) and Orphanet (https://www.orpha.net) catalog known rare disease genes. Complementing these resources, the AMELIE tool has been developed to curate knowledge of association between genes and phenotypes through extensive literature curation (Birgmeier et al. 2017). However, it is important to underline that these databases can rely on different semantics, and the variability of clinical terms when describing similar clinical features can lead to less accurate analyses. For genetic variants, several tools that incorporate prior biological knowledge are used to assess potential variant impacts including SIFT (Ng and Henikoff 2003), PolyPhen (Adzhubei et al. 2013), CADD (Kircher et al. 2014), LoFTEE (MacArthur et al. 2012), and M-CAP (Jagadeesh et al. 2016). As these tools and databases improve and expand, subsequent reanalysis of exomes continues to show improvement of diagnostic rate over time (Ewans et al. 2018; Nambot et al. 2018; Al-Nabhani et al. 2018; Wright et al. 2018b).

An inescapable limitation to exome sequencing is that it only characterizes the subset of the genome that encodes protein-coding genes. With rapid reductions in sequencing costs and a relatively unbiased survey of an individual's genetic variation, whole-genome sequencing (WGS) is a promising approach after the exome. For protein-coding regions, WGS provides the opportunity to find variants that are poorly captured using exome sequencing alone. In particular, WGS allows detection of structural variants more reliably (Meienberg et al. 2016; Stavropoulos et al. 2016). In a study aiming at comparing the diagnostic yield between conventional genetic tests and WGS on 103 patients with suspected genetic disorders, it was demonstrated that 18 diagnoses would not have been possible with exome sequencing alone as both structural and nonexonic variants were identified in disease associated genes (Lionel et al. 2018). It is expected that as costs decrease, WGS follow-up to exome-negative cases or as a first-line approach will become a new standard for clinical genomics (Stavropoulos et al. 2016; Lionel et al. 2018).

Whole-genome sequencing further provides access to a wealth of noncoding variants. Each individual carries approximately 30,000 rare variants across their genome (Li et al. 2017). A subset of these is expected to have important impacts on gene expression or alternative splicing. Recent estimates of the contribution of de novo mutation in fetal enhancer regions to neurodevelopmental disorders has identified that up to 3% of “exome-negative” cases could be explained by de novo mutations in regulatory regions (Short et al. 2018). This study was further notable in that it estimated that tens to hundreds of thousands of whole genomes would be required to comprehensively analyze the contributions of variants in noncoding elements to neurodevelopmental disorders. Exacerbating this challenge, there remains a lack of approaches to interpret increasingly complex scenarios in which coding and noncoding alleles act together to cause or modify a rare disease. The use of WGS as a routine will also require the development of robust methods to identify structural variations across control populations.

Regardless of DNA sequencing approach, it is estimated that the diagnostic rate using exome or genome sequencing is plateauing between 35% and 50% (Taylor et al. 2015; Wortmann et al. 2015; Clark et al. 2018; Powis et al. 2018). There is a need for other approaches to highlight the molecular signature of the disease and therefore target more efficiently the causal gene(s). A potential route is to focus on the consequences of pathogenic variants on different cellular products, among which are gene transcripts, proteins, or metabolites. By adding those layers of information, it is possible to identify aberrant products or activities that may further narrow the list of candidate genes and variants.

RNA-sequencing (RNA-seq), for example, has now become the gold standard to measure RNA levels and quantify transcript diversity. RNA-seq shows considerable diagnostic promise for rare diseases, as it provides a measurement of the consequences of both coding and noncoding variants on gene expression levels and alternative splicing (Byron et al. 2016). Under the assumption that only one or very few genes are impacted, RNA-seq makes it possible to detect and narrow investigation to the subset of genes with aberrant expression or splicing in affected individuals when compared to unaffected controls. There is further growing evidence that rare, genetic variants influence these aberrant events, providing further data to localize causal variants (Zhao et al. 2016; Li et al. 2017; Pala et al. 2017). By focusing in on a small set of outlier genes and their regulatory elements, there can be significant power advantages to detecting causal noncoding variants compared to studies that use only DNA-based sequencing data alone. Using the DDD model, we have estimated that pinpointing recurrent outlier activity for a specific regulatory element can substantially reduce the number of genomes required to associate de novo mutations with disease (Short et al. 2018; Fig. 2A). In the context of rare disease, recent applications of RNA-seq have aided in the diagnosis of multiple Mendelian diseases (Estivill 2015; UK10K Consortium et al. 2015; Xiong et al. 2015; Cummings et al. 2017; Kernohan et al. 2017; Kremer et al. 2017). Notably, it has helped to detect pathogenic splice variants in neuromuscular and mitochondrial diseases (Cummings et al. 2017; Kremer et al. 2017) some of which were previously undetected by exome sequencing alone (Kernohan et al. 2017). However, RNA-seq for “exome-negative” cases has only been used in very specific tissues and subsets of diseases so far and there remains a need to understand the potential applications of RNA-seq in the diagnosis of Mendelian diseases in a broader context.

**Figure 2.**
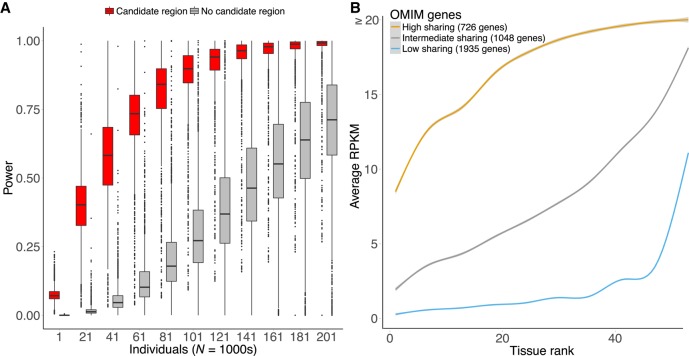
Using functional genomics to interpret rare diseases. (*A*) Power estimates for detecting a candidate regulatory region associated with a disease by the presence of recurrent de novo mutations. We compare power differences when there is a single candidate region (red) versus no candidate region (gray) using the model of Short et al. (2018). This demonstrates that additional biological knowledge of regions that are dysregulated in disease may significantly reduce the number of genomes required for their detection. (*B*) Ranked expression of OMIM genes across 53 tissues (GTEx v7). We used k-means to summarize expression data across all genes. Using three clusters, we show that we can separate our data as follow: high sharing, with high expression across all tissues (20% of OMIM genes); intermediate sharing, in which genes are expressed in a reasonable amount of tissues (28%); and low sharing, in which genes tend to be specifically expressed in a few tissues (52%). For 48% of OMIM genes, several tissues can be selected for RNA-seq analysis.

A potential limitation of RNA-seq (and other functional genomics assays) is that gene expression is dependent on environment, cell type, and state. This has direct consequences on the tissue to use for RNA-seq as a diagnostic tool for Mendelian diseases. Although any single tissue can be used for WGS, genes involved in a specific disease might not be expressed in a ubiquitous manner. Fortunately, the accumulation of RNA-seq studies in multiple tissues and databases provides an extensive resource to evaluate the expression status of a particular set of genes across a range of normal biological contexts (Su et al. 2004; Liu et al. 2008; Krupp et al. 2012; GTEx Consortium 2013; Kim et al. 2014, 2018; Uhlén et al. 2015; Papatheodorou et al. 2018). Use of these data increases the number of normal samples and can aid in differentiating between rare events that are random versus biological. The recent work of Cummings and collaborators (Cummings et al. 2017) took advantage of such an approach by comparing 180 control skeletal muscle samples from the GTEx Project to 50 cases with muscle disorders. They noted that many of their discoveries could not have been found in blood gene expression alone. With resources that describe cell type expression and gene/phenotype relationships, we expect that future computational approaches will further aid in diagnosis by predicting the relevant cell types to study. Indeed, analysis of gene expression shows 20% of OMIM genes are expressed across multiple tissues (Fig. 2B).

Induced pluripotent stem cells (iPSCs) constitute another promising avenue to overcome tissue specificity (Sterneckert et al. 2014; Zhang et al. 2015; Yamasaki et al. 2017). iPSCs provide access to expression of genes of interest not expressed in the patient's most accessible tissues. They have been shown to be extremely useful in the context of heart disease (Bellin and Mummery 2016) when derived to cardiomyocytes. However, iPSC lines are known to be heterogeneous (Lund et al. 2012; Germain and Testa 2017); thus, in the context of rare diseases, it is essential to separate expression variability due to the cell line from that due to the disease. There is also a trade-off to be found between the time and cost to develop iPSC lines for a patient and the likelihood that genes of interest are expressed and involved in the mechanisms of disease. The growing availability of iPSC transcriptome data from multiple individuals constitutes a new promising source to help mitigate these challenges and guide the decision of generating iPSCs for specific cases (Kilpinen et al. 2017; Panopoulos et al. 2017).

Regardless of the follow-up approach, a major challenge resides in the rarity of a patient's sample. In contrast to association studies for complex traits in which we can evaluate a trait across data from multiple individuals, patients with rare disease are often isolated cases. For gene expression data, when comparing data from one case to multiple controls, one needs to disentangle what is caused by inherent noise in the sample itself from the actual variation due to the disease. Analyses are further impacted by the sample imbalance of case versus controls. Indeed, sample imbalance has been shown to impact differential expression results (Yang et al. 2006). Robust methods have been developed for genome-wide association analyses from single-case exomes (Wilfert et al. 2016). There is a need in adapting those methods for other data modalities. In practice, increasing the number of controls from different studies not only helps to identify aberrant signals in an *N* = 1 sample but can run the risk of increasing batch effects by adding variability from individual studies. A crucial step when combining data sets is therefore to perform an adequate correction for those hidden batch effects, without compromising the relevant information in each sample (Stegle et al. 2012; Risso et al. 2014; Brechtmann et al. 2018).

The challenge of what to do next for “exome-negative” cases resides mainly in how effectively we can detect signal in either noisy or heterogeneous data. This is where adding layers of information and combining clues from different data modalities and statistical approaches can help narrow down from multiple candidate genes to a handful of meaningful ones. Gene expression is a promising next-step approach but is only one potential window to look for the effects of variants in exome-negative cases. Some variants, like missense, will not necessarily affect gene expression. Other scales of analysis, like proteome (Costanzo et al. 2017), metabolome (Gülbakan et al. 2016), or epigenome (Bjornsson 2015), have the potential to give access to consequences of those other types of variants. Adding up layers of information will drastically increase the probability of finding the causal gene. However, there will be a trade-off between large-scale functional genomics or more targeted approaches to confirm a suspected mechanism of action. The balance between processing time and cost can be poorly defined with respect to the value of added biological knowledge.

In the era of personalized medicine and high-throughput technologies, opportunities to diagnose the causes of rare diseases are abundant. Future efforts will take advantage of a growing ability to identify and classify different categories of variants. Intriguingly, current comparisons of genome and exome sequencing have shown similar diagnostic rates (Clark et al. 2018). However, much of the focus remains on protein-coding “loss-of-function” alleles, and new approaches are still required to identify different classes and categories of pathogenic variants, notably in conserved noncoding regions. Indeed, we have observed scenarios in which longer-read technologies are providing access to structural variants missed through exome sequencing alone (Merker et al. 2018) or family-based data help to identify inherited expression outliers (Pala et al. 2017). Furthermore, growing functional genomics resources such as TOPMed and GTEx provide data from thousands of healthy individuals and multiple biological contexts. Increasing the scale of these resources in natural and ex vivo contexts like iPSCs will eventually enable characterization of functional events that are present at frequencies of 1 in 10,000 or less. As such data types improve diagnostic yield in monogenic disorders, we expect that they will also provide new tools to identify the molecular causes of variable penetrance and oligogenic disorders. For “exome-negative” cases, with ongoing access to diverse data types complemented by data from healthy controls, our knowledge and ability to diagnose rare diseases only has room to grow.

## ADDITIONAL INFORMATION

### Acknowledgments

This work was supported by National Institutes of Health (NIH) grants R01HG008150 (NoVa) and U01HG009080 (GSPAC). We thank Matthew T. Wheeler, Craig Smail, and anonymous reviewers for their critical review of the manuscript.

### Competing Interest Statement

S.B.M. is on the SAB for Prime Genomics.
